# Emerging Fluorescent Molecular Tracers to Guide Intra-Operative Surgical Decision-Making

**DOI:** 10.3389/fphar.2019.00510

**Published:** 2019-05-14

**Authors:** Pieterjan Debie, Sophie Hernot

**Affiliations:** Laboratory for in vivo Cellular and Molecular Imaging (ICMI-BEFY/MIMA), Vrije Universiteit Brussel, Brussels, Belgium

**Keywords:** fluorescence-guided surgery, near-infrared fluorescence imaging, molecular imaging, intra-operative imaging, antibody-based fluorescent tracers

## Abstract

Fluorescence imaging is an emerging technology that can provide real-time information about the operating field during cancer surgery. Non-specific fluorescent agents, used for the assessment of blood flow and sentinel lymph node detection, have so far dominated this field. However, over the last decade, several clinical studies have demonstrated the great potential of targeted fluorescent tracers to visualize tumor lesions in a more specific way. This has led to an exponential growth in the development of novel molecular fluorescent contrast agents. In this review, the design of fluorescent molecular tracers will be discussed, with particular attention for agents and approaches that are of interest for clinical translation.

## Introduction

Surgery, in combination with or without chemo and/or radiotherapy, remains the most recommended treatment with curative intent for many localized tumors. In these types of surgeries, the primary goal is to attain complete removal of all cancerous tissue and obtain negative tumor resection margins. By minimizing the risk of leaving cancer cells behind, chances of recurrence are diminished and overall survival is improved (Javidfar et al., 2016; Orosco et al., 2018; Tringale et al., 2018).

As visual inspection through the surgeon's eyes (open surgeries) or through color video (laparoscopic interventions) is often insufficient, a technique for the detection of occult tumor lesions, accurate and real-time definition of tumor margins, and assessment of the presence of locoregional LN metastases is warranted. Such a technique would be a major step forward in preventing over- and under- treatment, and personalizing the surgical treatment of many cancer patients (Hentzen et al., 2018).

A recent trend is the usage of fluorescence imaging during surgery, which is a real-time, sensitive, contact-free, relatively cheap, and non-ionizing technique that can easily be implemented within the surgical routine. Fluorescence imaging can provide anatomical, functional, or molecular information after administration of a fluorescent contrast agent, either through direct real-time imaging of the surgical field, or by intra-operative optical specimen mapping (Nagaya et al., 2017; Zhang et al., 2017b).

The application of fluorophores emitting in the near-infrared (NIR) region of the light spectrum is favored as they enable detection of signals up to several millimeters of depth under the tissue surface (Weissleder and Ntziachristos, 2003; Kovar et al., 2007). Indocyanine green (ICG) and methylene blue (MB) are the only two NIR fluorescent dyes approved by the FDA, although the poor fluorescent properties of MB limit its use as fluorescent contrast agent (van Manen et al., 2018). ICG is a non-targeted hydrophobic dye that, once injected intravenously, aggregates to plasma proteins and is eliminated via the hepatobiliary route. Clinically-approved applications of ICG are determining cardiac output and hepatic function, and ophthalmic angiography (Soons et al., 1991; Iijima et al., 1997; Stanga et al., 2003). A broader range of applications has since been investigated, going from the use of ICG in reconstructive surgery for assessment of tissue perfusion (Holm, 2010), to sentinel lymph node mapping (Aoun et al., 2017; Chand et al., 2018), ureter visualization (Siddighi et al., 2014), and in some cases, tumor imaging (Van Der Vorst et al., 2013; Liberale et al., 2017; Nakaseko et al., 2018). ICG tumor uptake is reliant on the enhanced permeability and retention (EPR) effect, which, while effective in mouse models, has seen only limited applicability in the human situation due to differences in tumor growth and development (Nichols and Bae, 2014; Danhier, 2016), and has been demonstrated to lead to false-positive results for image-guided surgery (Tummers et al., 2015).

As fluorescent dyes by themselves generally lack tumor specificity, molecular targeted fluorescent agents have been introduced. Targeted tracers consist of a fluorophore chemically conjugated to a targeting moiety, the latter possessing binding affinity for a specific cancer-associated molecular target or biomarker. Targeted tracers that are always fluorescent, are referred to as “always-on” targeted tracers. In this review the different parameters to the design of these tracers will be discussed, from the level of target selection, to the choice of targeting moiety, fluorophore, and conjugation strategy. Alternatively, probes can be designed around mechanisms that cause fluorescence to be quenched until activation. This can be used for the development of “activatable” or “smart” fluorescent probes. An overview of the different types of activatable tracers will also be provided.

## Molecular-Targeted Fluorescent Tracers

### Conjugatable NIR Fluorophores

In fluorescence image-guided surgery (FIGS) research and practice, fluorophores operating in the NIR region are most often used, because of superior imaging characteristics compared to light in the visible spectrum. More specifically, the lower background and improved tissue penetration -a consequence of diminished scattering, endogenous fluorophore absorption and autofluorescence- makes NIR light more suited (Weissleder and Ntziachristos, 2003; Kovar et al., 2007). The NIR domain applicable for *in vivo* optical imaging can actually be separated in two distinct areas: the NIR-I (650–900 nm) and NIR-II window (1,000–1,700 nm).

#### The NIR-I Window

**Figure 1:**
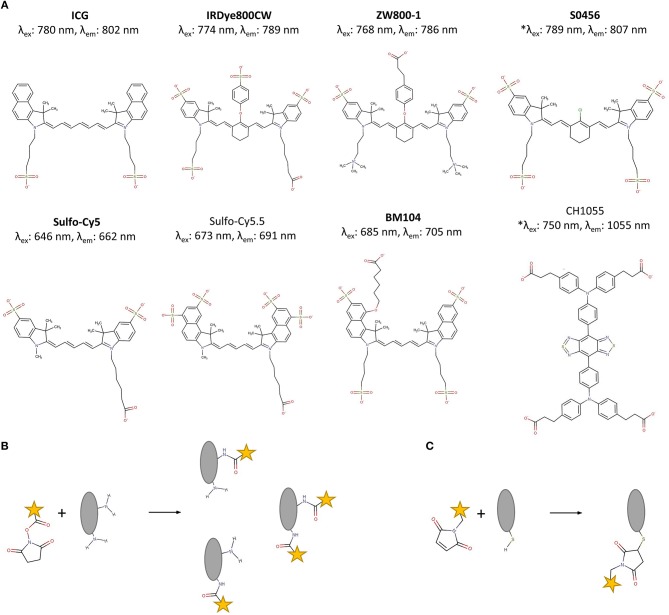
**(A)** Deprotonated structures of IRDye800CW, the most used conjugated fluorophore in clinical trials, and other commonly used NIR fluorophores for FIGS, with values for wavelengths of maximal excitation and emission in PBS. The names of all clinically evaluated fluorophores are displayed in bold. ^*^S0456 values measured in MeOH, CH1055 values measured for the PEGylated form. **(B)** Random conjugation of an NHS-functionalized fluorophore (yellow) on the primary amines of a protein targeting ligand (gray). A mix with fluorophores conjugated to different positions is obtained. **(C)** Site-specific conjugation of a maleimide-functionalized fluorophore (yellow) on a C-terminal sulfhydryl residue of a protein targeting ligand (gray). The final tracer has a single fluorophore per targeting moiety. Chemical structures were drawn using MarvinSketch 19.2 (ChemAxon, Budapest, Hungary).

In the NIR-I region, a wide choice of fluorophores is available for conjugation. Appropriate fluorophores should be (photo)stable *in vitro* and *in vivo*, be soluble in aqueous environments, and their extinction coefficient and quantum yield should be as high as possible to maximize brightness. For the labeling of proteins and small molecule ligands for FIGS, cyanine-based fluorescent dyes are by far the most widely investigated (Figure 1A). Cyanine dyes typically consist of two heterocyclic nitrogen-containing rings connected by a polymethine bridge. Extension of the polymethine bridge to penta- and heptamethines, red-shifts the absorption and emission of the dyes (Yi et al., 2014; Zhang et al., 2017b). In comparison to ICG, the most commonly used cyanine dyes have been rendered less hydrophobic by the inclusion of several charged groups such as sulfonic acids (e.g., sulfo-Cy5, sulfo-Cy7) in their structure resulting in less non-specific protein interactions *in vivo* (Mujumdar et al., 1993). In the synthesis of heptamethine dyes, it has been found that the length of the polymethine chain has a negative effect on the chemical stability, as well as the photochemical properties of the dye. This has resulted in the integration of a central cyclohexenyl ring into the structure, which improves stability and fluorescence quantum yield through the increased rigidity of the molecular structure (Tarazi et al., 1998).

IRDye800CW (Figure 1A) is the most applied dye for the design of targeted FIGS tracers. IRDye800CW is primarily cleared via the kidneys when intravenously injected, with some liver uptake, though much less than for ICG (Marshall et al., 2010). An alternative development was that of the zwitterionic dye ZW800-1 (Figure 1A). Due to balanced surface charges on its structure, this fluorophore interacts very little with serum proteins, and is almost exclusively cleared by the kidneys (Choi et al., 2011). However, the ether linkage on the meso carbon at the core of this dye has been found to be unstable *in vivo*, leading to a decrease of the fluorescent signal over time. This issue was resolved by replacing this ether linkage with a carbon-carbon bond (Hyun et al., 2014). Yet, this modification comes at the cost of increased formation of H-dimers, which are caused by stacking of the fluorophores (Ogawa et al., 2009). Both IRDye800CW and ZW800-1 have, due to their renal clearance, also been proposed for ureter imaging (Choi et al., 2011; Korb et al., 2015).

Other cyanine dyes that have been investigated (pre)clinically for FIGS include the pentamethine dyes BM104, Dy676, IRdye650, IRDye680RD, and Alexa Fluor 680 (AF680), and the heptamethine dyes FNIR-Z-759, Xenolight 770CF, S0456, LS288, and ZWCC. Even ICG is sometimes used as a conjugatable fluorophore in always-on targeted tracers (Juhl et al., 2016), though it is most often incorporated in activatable targeted tracers, as aggregation of the dye to the structure of the targeting moiety will cause quenching, until the tracer is metabolized (Kobayashi et al., 2016). It is possible to use NIR dyes based on other backbones, such as BODIPYs, squaraines, rhodamines and porphyrins, however, these are not often used for FIGS, given their either excessive hydrophobicity or poor photochemical properties (Zhang et al., 2017b). The spectral properties of the chosen dye are however interdependent with the excitation light source and emission collection specifications of the camera system used during imaging.

#### The NIR-II Window

Imaging in the NIR-II window has further advantages related to autofluorescence and scattering compared to the NIR-I window, though absorption by water is slightly higher at these wavelengths. NIR-II imaging, however, is difficult due to low quantum yields, poor water solubility and general lack of availability of biocompatible fluorophores (Hong et al., 2017). Only recently, Antaris et al. described the synthesis of the biocompatible and renally cleared NIR-II organic fluorophore CH1055 (Figure 1A) and its use for targeted tumor imaging after conjugation with an anti-Epidermal Growth Factor Receptor (EGFR) affibody-molecule (Antaris et al., 2016). This field may gain more traction in the near future (Jin, 2019).

### Hybrid Nuclear and Fluorescent Labels

While the use of NIR fluorescent dyes has led to increased signal-to-background ratios and better overall depth penetration up to several millimeter in tissue, deeper lying, and hidden lesions would still be missed. To this end, the combination of NIR fluorescence and nuclear techniques in a single tracer may be attractive. The nuclear component can be used for pre-operative imaging, as well as for intra-operative guidance using a gamma-detecting probe (coarse navigation), at which point, fluorescence would provide high resolution visual guidance for precise resection (Van Leeuwen et al., 2016). Integration of both modalities onto a tracer can occur in several ways. Conjugation of the fluorophore and chelator for radioactive labeling at different positions is one option, either through co- or sequential incubation of the targeting ligand with the chosen fluorophore and chelator (Lütje et al., 2014b). Several preclinical hybrid single photon emission tomography (SPECT)/fluorescent tracers combining IRDye800CW and ^111^In have been prepared in this manner, including the antibodies BIWA against CD44v6 (Odenthal et al., 2018) (**Figure 3A**), MN-14 and Labetuzumab against carcinoembryonic antigen (CEA) (Rijpkema et al., 2014; Hekman et al., 2017), and D2B against prostate specific membrane antigen (PSMA) (Lütje et al., 2014a). This technique has also been applied clinically, with the carbonic anhydrase 9 (CAIX) specific antibody Girentuximab (Hekman et al., 2016, 2018). The same manner of conjugation was used to prepare preclinical positron emission tomography (PET)/NIR tracers by labeling the anti-CD146 antibody YY146 with IRDye800CW and ^89^Zr (Hernandez et al., 2016), the anti-prostate stem cell antigen (PSCA) minibody A11cMb with a Cy5.5 dye and ^89^Zr/^124^I (Tsai et al., 2018), or the anti-prostate stem cell antigen (PSCA) diabody A2cDb with IRDye800CW and ^124^I (Zettlitz et al., 2018).

In another integration strategy, the fluorophore and the radiolabel can be combined into a single structure. This is advantageous since a more homogenous tracer is obtained, whose biodistribution can be accurately determined via radioactivity. Furthermore, this is often the only possibility when working with small peptides or molecules, as multiple attachment points may not be available. However, such structures are more complex to design, as opposed to the more straightforward separate labeling procedure. A urokinase-type plasminogen activator receptor (uPAR) specific antibody, called ATN-658, was conjugated with both ZW800-1 and a chelator for ^111^In labeling through a single structure called a “multifunctional single attachment point” (MSAP) that bore both labels (Boonstra et al., 2015c, 2017). The MSAP-strategy was also used to combine an ^111^In/chelator complex and Cy5.5 fluorophore for conjugation to either an integrin binding RGD peptide or the anti-TZ14011 peptide targeting chemokine receptor 4 (CXCR4) (Kuil et al., 2011; Buckle et al., 2013). Additional dual labeling structures have been explored, for example, the construction of a dual-modality linker combining Cy5 and ^18^F on the diabody A2cDb (Zettlitz et al., 2019), or for the preparation of a CH1055-^68^Ga-RGD tracer (Sun et al., 2018), ^68^Ga-IRDye650/IRDye800CW conjugated bombesin antagonists (Zhang et al., 2017a; Li et al., 2018), or a Cy5-labeled anti-PSMA KuE peptide with a chelator for ^68^Ga or ^177^Lu labeling for PET or SPECT/targeted radionuclide therapy (TRNT), respectively (Schottelius et al., 2018).

### Labeling Strategies

Fluorophores (and by extension, hybrid labels) can be linked to the targeting moiety using a range of different conjugation chemistries. As opposed to small molecules and peptides that are generally more tolerant toward harsher conditions such as the use of organic solvents and high temperatures, the conjugation of fluorophores to larger protein-based moieties is restricted to softer conditions. The most commonly applied strategy to this end is the formation of an amide bond between primary amines, and an activated carboxylic acid (often activated via a N-hydroxysuccinimide (NHS)-ester, Figure 1B) (Toseland, 2013). While every polypeptide contains a primary amine at its N-terminus, this method is often applied for random conjugation on lysine residues. In larger protein structures, the abundancy of accessible lysines makes the degree of conjugation and the exact positioning of fluorophores difficult to control, resulting in a heterogenous tracer mixture. Furthermore, having many fluorophores per targeting ligand can lead to self-quenching effects (reduced brightness) and if the fluorophores are conjugated in or close to the antigen-binding region, they may negatively impact the binding capacities of the tracer (Debie et al., 2017). This can especially be a problem for smaller compounds, though it has been demonstrated that even the binding efficiency of full antibodies can be affected (Vira et al., 2010; Szabó et al., 2018).

Site-specific labeling methods resulting in homogenous tracer preparations in which the fluorophore is only attached to a specific, pre-determined location situated away from the antigen-interaction site, may offer a solution. The formation of a thio-ether bond between a sulfhydryl residue and a maleimide functionalized fluorophore is an often-employed strategy in FIGS (Figure 1C). To this end, a free cysteine residue is incorporated in the targeting moiety's structure at the location of predilection, such as the C- or N-terminus of protein scaffolds, peptides and small antibody fragments (Sun et al., 2005; Massa et al., 2014). Antibodies and larger fragments such as minibodies and diabodies can furthermore be partially reduced, freeing the sulfhydryl residues of more exposed disulfide bridges for conjugation (Olafsen et al., 2004; Sonn et al., 2016; Tsai et al., 2018; Zettlitz et al., 2018; Zhang et al., 2018). Many other site-specific methods have gained popularity over the last years, such as the azide-alkyne cycloaddition-click chemistry (Li et al., 2014), enzymatic modification of peptide tags (e.g., transglutaminase-, sortase-, or intein-mediated conjugation) (Massa et al., 2016a), incorporation of unnatural amino acids for biorthogonal conjugation or the use of linking peptides such as the SPY/SPYcatcher method (Massa et al., 2016b; Alam et al., 2018). While these techniques are promising for the future development of FIGS tracers, to date, they have not yet been applied in clinical tracers, and have seen only limited development in preclinical FIGS research.

### Impact of Fluorophore and Conjugation on the Tracer's Pharmacokinetics

**Figure 2:**
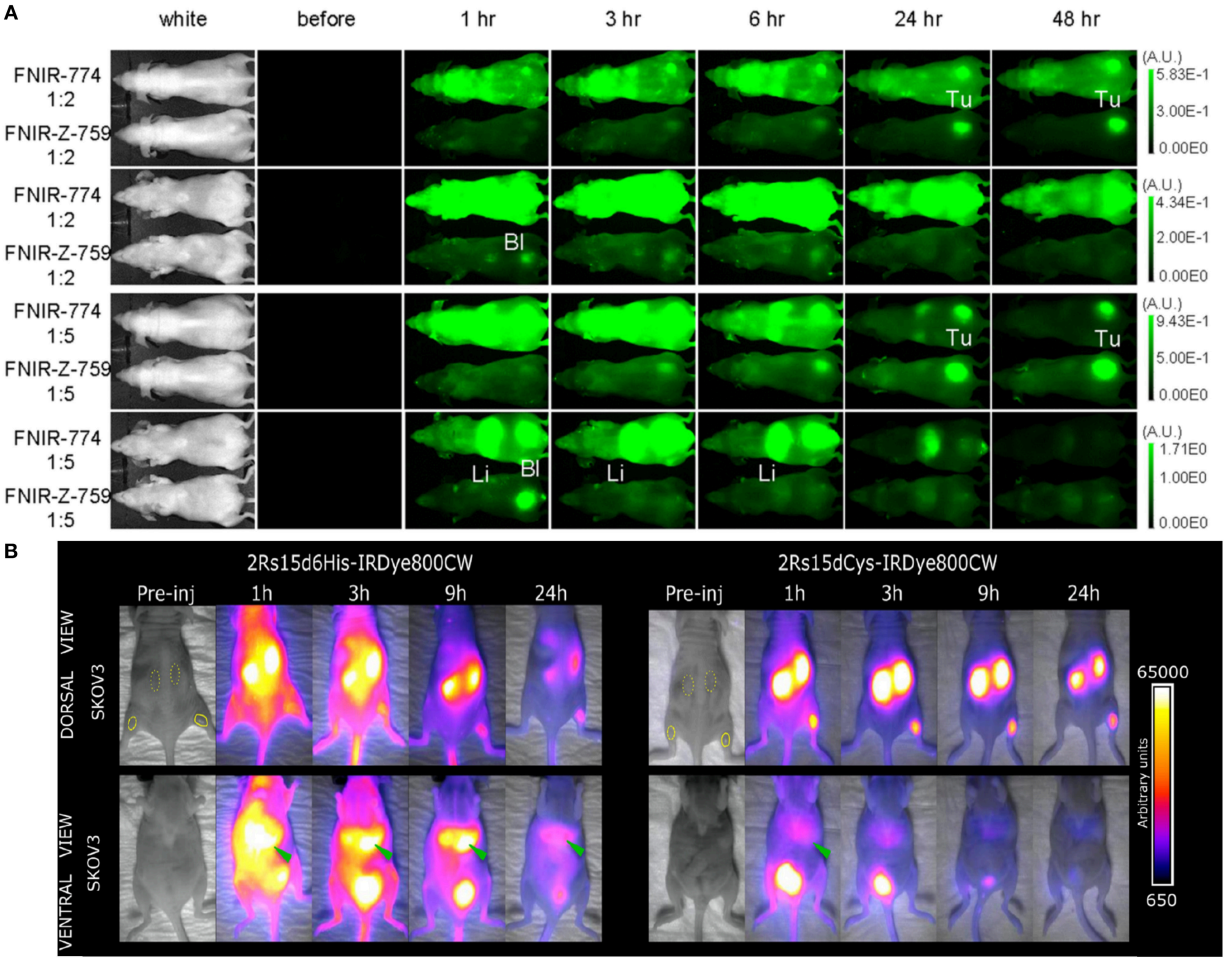
Examples of the influence of the fluorescent dye and conjugation strategy on the pharmacokinetic profile of tracers. **(A)** Dorsal and ventral images of the *in vivo* biodistribution of panitumumab randomly conjugated (2:1 or 5:1 fluorophore vs. antibody ratio) with a highly negatively charged (FNIR-Z-774) or zwitterionic (FNIR-Z-759) fluorophore. The zwitterionic fluorophore promotes renal elimination and faster clearance, while the negatively charged fluorophore causes hepatic clearance and higher background fluorescence. Adapted with permission from Sato et al. (2016b). Copyright (2016) American Chemical Society. **(B)** Dorsal and ventral images of biodistribution of anti-HER2 nanobody 2Rs15d either randomly or site-specifically conjugated to IRDye800CW. Random conjugation promotes increased hepatic clearance and higher background fluorescence of the tracer, as opposed to the site-specifically conjugated nanobody that was mainly renally cleared. Adapted with permission from Debie et al. (2017). Copyright (2017) American Chemical Society.

Conjugation of a fluorescent dye to a targeting ligand can possibly alter the pharmacokinetics of the ligand. The physicochemical properties of the dye (e.g., hydrophilicity and global/local charge distributions), the degree of conjugation, as well as the chosen conjugation method all play a role in the general biodistribution of the FIGS tracer. It has been observed that after labeling with IRDye800CW, tracers most often exhibit more extensive non-specific targeting *in vivo*, including higher liver uptake. This is likely caused by the interaction of IRDye800CW with serum proteins. Further hepatic metabolization of the tracer can in addition be a source of intestinal background signal which could be contrast-limiting in abdominal applications (Choi et al., 2011, 2013; Debie et al., 2017). This is in accordance with findings for other heptamethine fluorophores, where a high number of negative charges on the fluorophore -as is the case for IRDye800CW- are seen to cause partial hepatobiliary clearance (Sato et al., 2016b) (Figure 2A). Other than merely causing increased background signals, interaction with serum proteins will furthermore cause a change in the imaging window of the tracer. While this effect is already observed for long-circulating antibodies (Sato et al., 2016b; Cilliers et al., 2017), the impact on smaller compounds that usually show fast pharmacokinetics and renal clearance can be considerably more pronounced, in some cases delaying the optimal imaging window from a few hours to up to a day or longer. Indeed, increased liver uptake and delayed optimal tumor-to-background ratios (TBR) were observed for randomly IRDye800CW conjugated nanobodies (Figure 2B), scFv's, Fab fragments and diabodies (Yang et al., 2014; Boonstra et al., 2015b; Debie et al., 2017; El-Sayed et al., 2018; Boogerd et al., 2019). Interestingly, the gravity of this effect has been found to also be related to the used conjugation chemistry. For instance, we found that the pharmacokinetics of site-specifically IRDye800CW or IRDye680RD conjugated nanobodies normalized compared to randomly-conjugated nanobodies (Figure 2B). These nanobody-based tracers could be used for high contrast imaging as early as 1 h post-injection (Kijanka et al., 2016; Debie et al., 2017). Of note, while not directly compared to their randomly-conjugated analog, affibodies expressing a C-terminal cysteine and conjugated at this position with IRDye800CW also enabled imaging at the expected early timepoints (Sexton et al., 2013; de Souza et al., 2017; Ribeiro de Souza et al., 2018).

With the aim of optimizing the pharmacokinetic profile of tracers, the chemical design of fluorophores can be systematically modified. Variations in the overall charge, total number of charges and hydrophilicity of a Cy5 dye have led to a hybrid cRGD-tracer with improved properties regarding non-specific background signals, renal elimination, and tumor uptake (Bunschoten et al., 2016). A similar strategy was applied for the optimization of the cRGD peptide as well as a KUE anti-PSMA peptide, with heptamethine fluorophores. However, conclusions of such studies on the most optimal design of fluorophores for peptide conjugation are difficult to generalize as the obtained results are also highly dependent on the physicochemical properties of the peptide itself (Choi et al., 2013; Bao et al., 2017). It is therefore becoming evident, that selection of an appropriate fluorophore and labeling strategy is an essential part of the fluorescent tracer design. For protein-based ligands such as antibodies, antibody-fragments, and scaffold proteins general properties and guidelines concerning the conjugation of certain fluorophores can be established, whereas in small peptides and molecules optimization in the design of new conjugates may be required on an individual basis.

### Targets for FIGS With Molecular-Targeted Tracers

In the current preclinical and clinical FIGS studies, a wide variety of biomarkers have been investigated as potential targets. Extracellular molecules enable targeting with non-cell penetrating ligands, and cell-membrane bound biomarkers are often preferred, as diffusion of secreted targets will reduce the sharpness of imaging. Biomarkers overexpressed by the cancer cells themselves are popular as they are already widely used for targeted therapeutic or nuclear imaging applications (Boonstra et al., 2016). Targets having shown high-level and quite general overexpression within specific cancer types include folate receptor α (FRα), which is mainly known for ovarian cancer (Kalli et al., 2008), CEA in colorectal and pancreatic cancer (van Oosten et al., 2011; de Geus et al., 2016), and PSMA in prostate cancer (Perner et al., 2007). One of the most highly overexpressed biomarkers is the breast cancer marker human epidermal growth factor 2 (HER2). While high contrast in HER2 overexpressing cells will likely be achieved, the fact that this marker is only overexpressed in a small subset of patients can be problematic in the sense that pre-operative screening would be required (Harbeck and Gnant, 2017). In order to generate more widely applicable FIGS tracers, the search for more general tumor markers is ongoing. Targets such as EGFR, carbonic anhydrase 9 (CAIX) and the epidermal cell adhesion molecule (EpCAM) have been suggested and explored to this end (Boonstra et al., 2015a, 2016).

Another strategy lies in the targeting of the tumor stroma, instead of only the tumor cells. This has several distinct advantages, namely, stroma is present on the tumor periphery/invasive edge and is part of a general physiological process, implying the presence of cancer-type independent markers, such as markers associated with infiltration of angiogenesis, tumor associated immune cells, or fibroblasts (Boonstra et al., 2015a). Examples of potential stromal targets include the soluble, cell-released vascular endothelial growth factor (VEGF-A) as marker for angiogenesis or the fibroblast activation protein alpha (FAP-α) for fibroblast targeting. Several markers also share stromal and tumor cell expression, and may as such be especially interesting. Examples are uPAR and integrins such as α_v_β_3_ (Boonstra et al., 2015a; Hamidi and Ivaska, 2018). In all cases, constitutive expression in normal or fibrotic tissue could be a limiting factor as it will cause on-target, off-tumor uptake. Moreover, one should always take intra-tumoral heterogeneity into consideration (Pogue et al., 2018).

### Targeting Ligands

As for targeting moieties, a host of possible types of molecules are available. These range from full IgG antibodies, to antibody fragments, scaffold proteins, peptides, and small molecules. A comprehensive overview of the different targeting ligands that have been investigated preclinically and clinically in the context of FIGS is given in Table 1.

**Table 1:** Comprehensive overview of potential targeted “always-on” FIGS tracers.

**Tracer type**		**Compound name**	**Molecular target**	**Imaging target**	**Reporter fluorophore(s)**	**References**	**Remarks**
Antibodies/Antibody fragments	Antibody	Girentuximab	CAIX	Renal cancer^C, Ev^	IRDye800CW	Hekman et al., 2016, 2018	Multimodal, Clinical trial
		MabCAIX		Breast cancer^O^	IRDye800CW	Van Brussel et al., 2013	
		YY146	CD146	Hepatocellular cancer^S, O^	ZW800-1	Hernandez et al., 2016	Multimodal
		BIWA	CD44v6	Head and Neck cancer^O^	IRDye800CW	Odenthal et al., 2018	Multimodal
		MN-14	CEA	Colorectal cancer^S, Ip^	IRDye800CW	Rijpkema et al., 2014	Multimodal
		SGM-ch511 (SGM-101)		Colorectal cancer^C, O, Ip, M^, Pancreatic cancer^C, O^	BM104	Gutowski et al., 2017; Boogerd et al., 2018; Hoogstins et al., 2018	Clinical trial
		Labetuzumab		Colorectal cancer^S, O^	IRDye800CW	Hekman et al., 2017	Multimodal
		hM5A		Pancreatic cancer^S, O, Pdx^	IRDye800CW	Lwin et al., 2018b,c	
		Cetuximab	EGFR	Breast cancer^S^, Head and Neck cancer^C, S, O^, Pancreatic cancer^C^, Fibrosarcoma^S^	IRDye800CW	Day et al., 2013b; Korb et al., 2014; De Boer et al., 2015; Warram et al., 2015, 2016; Rosenthal et al., 2016, 2017; Moore et al., 2017; Gao et al., 2018a; Prince et al., 2018; Tummers et al., 2018b	Clinical trial
		Panitumumab		Breast cancer^S^, Colorectal cancer^S^, Head and Neck cancer^C, O^, glioma^S, O^, Skin cancer^S, O^	IRDye800CW, FNIR-Z-759	(Heath et al., 2012; Day et al., 2013a,b; Gong et al., 2014; Korb et al., 2014; Sato et al., 2016a,b; Gao et al., 2018a,b; Marston et al., 2019; van Keulen et al., 2019)	Clinical trial
		mAb 7.4	EpCAM	Prostate cancer^O^	IRDye800CW	Zhu et al., 2013	
		323/A3		Colorectal cancer^O^, Breast cancer^O, Ip^, Head and Neck cancer^O^	IRDye800CW	van Driel et al., 2016	
		Trastuzumab	HER2	Breast cancer^S^, Gastric cancer^Ip^, Ovarian cancer^S^	IRDye800CW	Terwisscha van Scheltinga et al., 2011; Korb et al., 2014	
		Tocilizumab	IL-10	Breast cancer, Melanoma^S, M^	IRDye800CW	Day et al., 2013a; Korb et al., 2014	
		mAb62	K_v_10.1	Melanoma^S^	Cy5.5	Napp et al., 2016	
		D2B	PSMA	Prostate cancer^S^, Colorectal cancer^M^	IRDye800CW	Lütje et al., 2014a	Multimodal
		Bevacizumab	VEGF-A	Breast cancer^C, S^, Colorectal cancer^C^, Gastric cancer, Ovarian cancer^S^, Skin cancer^S, O^	IRDye800CW	Terwisscha van Scheltinga et al., 2011; Day et al., 2013a; Korb et al., 2014; Harlaar et al., 2016; Lamberts et al., 2017	Clinical trial
		DC101	VEGFR-2	Fibrosarcoma^S^	IRDye800CW	Prince et al., 2018	
		ATN-658	uPAR	Colorectal cancer^S, O^, Head and Neck cancer^O^	ZW800-1	Boonstra et al., 2015c, 2017	Multimodal
	(Fab)_2_	TRC-105 & ALT-836 heterodimer	CD105/TF	Pancreatic cancer^S^	ZW800-1	Luo et al., 2017	
	Minibody	A11 (c)Mb	PSCA	Prostate cancer^S, O, M, Im^	Cy5.5, IRDye800CW	Tsai et al., 2018; Zhang et al., 2018	Multimodal
	Diabody	anti-HER3 diabody	HER3	Pharyngeal cancer^S^	IRDye800CW	Alam et al., 2018	
		A2cDb	PSCA	Prostate cancer^S^, Pancreatic cancer^Pdx^	Cy5, IRDye800CW	Sonn et al., 2016; Zettlitz et al., 2018, 2019	Multimodal
	Fab	anti-CEA	CEA	Colorectal cancer^S^	Dy676	Lisy et al., 2008	
		VB5-845d	EpCAM	Colorectal cancer^S^, Breast cancer^O^	IRDye800CW	Boogerd et al., 2019	
	ScFv	ssSM3E	CEA	Colorectal cancer^S, O^, Pancreatic cancer^O^	IRDye800CW	Boonstra et al., 2015b	
		ScFvEGFR	EGFR	Breast cancer^O^	IRDye800CW	Yang et al., 2014	
		scFvD2B	PSMA	Prostate cancer^O^	XenoLight 770CF	Mazzocco et al., 2016	
		3E8.scFv.Cys	TAG-72	Colorectal cancer^O^	IRDye800CW	Gong et al., 2018	
	Nanobody	B9	CAIX	Breast cancer^O^	IRDye800CW	Kijanka et al., 2016	
		7D12, 7D12-9G6	EGFR	Head & Neck cancer^O^	IRDye800CW	Van Driel et al., 2014	
		2Rs15d	HER2	Ovarian cancer^S, Ip^	IRDye800CW, IRDye680RD	Debie et al., 2017, 2018	
		11A1		Breast cancer^S^	IRdye800CW	Kijanka et al., 2013, 2016	
Protein Scaffolds	Centyrin	83v2Cys	EGFR	Lung cancer^S^	S0456	Mahalingam et al., 2017	
	Affibody	Z03115-Cys	EGFR	Glioma^O^	IRDye800CW	Sexton et al., 2013; de Souza et al., 2017; Ribeiro de Souza et al., 2018	
		Z_EGFR:1907_		Skin cancer^S^	Cy5.5, AF680, IRDye800CW	Miao et al., 2011; Qi et al., 2012	
		Z_HER2_	HER2	Breast cancer^S^	DyLight-750	Zielinski et al., 2012	
	Cystine knottin	R01-MG	Integrin α_v_β_6_	Pancreatic cancer^S, O, Tg^	IRDye800CW	Tummers et al., 2018a	
		Chlorotoxin	ANXA2, MMP-2	Breast cancer^C^, Glioma^S, O^, Head and Neck cancer^O^, Spontaneous canine tumors	ICG, Cy5.5	Veiseh et al., 2007; Butte et al., 2014; Kittle et al., 2014; Fidel et al., 2015; Baik et al., 2016; Dintzis et al., 2018	Clinical trial
Peptides		cRGD	Integrins α_v_β_3_, α_v_β_5_ and α_v_β_6_	Breast cancer^O^, Colorectal cancer^S, O^, Gastric cancer^S, Ip^, Glioma^S, O^, Head and Neck cancer^O^, Lung cancer^M^, Skin cancer^S^, Pancreatic cancer^O^	ICG, Cy5, Cy5.5, ZW800-1, IRDye800CW, CH1055^*^	Huang et al., 2012; Choi et al., 2013; Verbeek et al., 2014; Cheng et al., 2016; Handgraaf et al., 2017; Liu et al., 2018; Sun et al., 2018	^*^NIR-II, Multimodal
		NGR	CD13	Glioma^S, O^, fibrosarcoma^S^	Cy5.5	Li et al., 2014; Liu et al., 2018	
		Ac-TZ14011	CXCR4	Breast cancer^S^	CyAL-5.5b	Kuil et al., 2011; Buckle et al., 2013	Multimodal
		EGF	EGFR	Head and Neck cancer^O^, glioma^S^	IRDye800CW	Keereweer et al., 2012; Gong et al., 2014	
		TM1	GRPR	Head and Neck cancer^O^	IRDye680RD	Suganya et al., 2016	
		G-pip-Sta-BBN, GSG-Sta-BBN,6Ahx-Sta-BBN		Prostate cancer^S^	AF750	Xu et al., 2018	
		BBN[7-14]NH2		Glioma^C, O^, Prostate cancer^O^	AF680, IRDye800CW	Cai et al., 2013; Li et al., 2018	Multimodal Clinical trial
		BBN[6-14]NH2		Prostate cancer^S^	IRDye650	Zhang et al., 2017a	Multimodal
		OTL78 (DUPA)	PSMA	Prostate cancer^S^	S0456	Kularatne et al., 2018	
		KuE		Prostate cancer^S^	Cy5, ZW800+3C	Bao et al., 2017; Schottelius et al., 2018	Multimodal
		Glu-urea-Lys-HBED-CC		Prostate cancer^S^	IRDye800CW, DyLight800	Baranski et al., 2017	Multimodal
		PSMA-1		Prostate cancer^S, O^	Cy5.5, IRDye800CW	Wang et al., 2014	
		AE105	uPAR	Glioma^O^, Head and Neck cancer^O^	ICG, CH1055	Christenen et al., 2017; Kurbegovic et al., 2018	
		ATF		Breast cancer^O^, Pancreatic cancer^O^	Cy5.5, NIR-830	Yang et al., 2014	
Small molecules		Hypoxyfluor-1	CAIX	Colorectal cancer^S^	S0456	Mahalingam et al., 2018a	
		Z-360	CCK2R, CCK2i4svR	Transfected HEK 293T^S, M^	LS288	Wayua and Low, 2014	
		OTL38	FR	Cervical cancer^S^, Lung cancer^C^, Ovarian cancer^C^	S0456	Hoogstins et al., 2016; Keating et al., 2017; Mahalingam et al., 2018b; Predina et al., 2018	Clinical trial
		EC17		Breast cancer^C^, Ovarian cancer^C^, Renal cancer^S, C^	Fluorescein	van Dam et al., 2011; Guzzo et al., 2016; Tummers et al., 2016	Clinical trial
		2-DG	GLUT1	Head and Neck cancer^O^	IRDye800CW	Keereweer et al., 2012	
		BOEPL	LHRH-R	Breast cancer^S^, Endometrial cancer^S^, Ovarian cancer^S^	S0456	Roy et al., 2019	
		L-733,060	NK1R	Transfected HEK 293T^S^	LS288	Kanduluru et al., 2016	
		YC-27	PSMA	Prostate cancer^S, O^	IRDye800CW	Chen et al., 2009; Neuman et al., 2015	
		IY-IY	TrkC	Breast cancer^S^	ZWCC	Yang et al., 2018	

* *C, In clinical trial; Ev, Ex vivo human tumor samples; Ip, small animal intraperitoneally disseminated tumor model; M, small animal metastatic tumor model; O, small animal orthotopic tumor model; Pdx, small animal patient-derived xenograft tumor model; S, small animal subcutaneous tumor model; Tg, small animal transgenic spontaneous tumor model*.

*ANXA2, annexin a2; CAIX, carbonic anhydrase 9; CCK2R, cholecystokinin 2 receptor (CCKi4svR is a splice variant); CEA, carcinoembryonic antigen; CXCR4, C-X-C chemokine receptor type 4; EGFR, epidermal growth factor receptor; EpCAM, epithelial cell adhesion molecule; FR, folate receptor; GLUT1, glucose transporter 1; GRPR, gastrin releasing peptide receptor; HER2/3, human epidermal growth factor receptor 2/3; LHRH-R, Luteinizing hormone releasing hormone receptor; IL-10, interleukin 10; MMP-2, matrix metalloproteinase 2; NK1R, neurokinin 1 receptor; PSCA, prostate stem cell antigen; PSMA, prostate specific membrane antigen; TAG-72, tumor-associated glycoprotein 72; TF, tissue factor; TrkC, tropomyosin receptor kinase C; uPAR, urokinase plasminogen activator receptor; VEGF-A, vascular endothelial growth factor A; VEGFR-2, Vascular endothelial growth factor receptor 2*.

Antibodies are large (150 kDa) proteins that bivalently attach to their molecular target with high affinity and specificity. The large size of antibodies, as well as the presence of an Fc-domain bestow a long blood half-life and limit tumor penetration. Indeed, as is known from nuclear molecular imaging, the use of antibodies as targeted fluorescent contrast agents for FIGS necessitates extended waiting periods of several days to obtain sufficient target-specific contrast (Freise and Wu, 2015). Antibody-based FIGS tracers represent the largest group of currently evaluated compounds as monoclonal antibodies against a wide variety of targets are readily available and can be straightforwardly repurposed following fluorophore conjugation. For example, the therapeutic EGFR targeting antibodies Cetuximab and Panitumumab, and the anti-VEGF-A antibody Bevacizumab have been, after labeling with IRDye800CW, broadly explored in the context of FIGS in a range of pathologies such as head and neck cancer, colorectal cancer, breast cancer, pancreatic cancer, and glioblastoma (Heath et al., 2012; Day et al., 2013a,b; Gong et al., 2014; Korb et al., 2014; De Boer et al., 2015; Warram et al., 2015, 2016; Rosenthal et al., 2016, 2017; Moore et al., 2017; Gao et al., 2018a,b; Prince et al., 2018; Tummers et al., 2018b; van Keulen et al., 2019). Early phase clinical trials demonstrated the feasibility of intraoperative lesion detection and this was confirmed by histological evidence. The anti-CEA compound SGM-101, consisting of the novel antibody SGM-ch511 and the fluorophore BM104 (Figure 1A) (the NHS-activated version of BM104 is called BM105) is currently being evaluated in a phase III clinical trial for colorectal cancer. In addition, the potential of many more NIR-labeled antibodies (most often labeled with IRDye800CW) has been investigated in a variety of subcutaneous, orthotopic, and metastatic mouse models, including the anti-(human) EpCAM antibody 323/A3 (van Driel et al., 2016) or anti-uPAR antibody ATN-658 (Boonstra et al., 2017) for colorectal, head and neck, and/or breast cancer.

To achieve more rapid pharmacokinetics, antibody fragments, obtained through protein engineering, can be considered. Minibodies, consisting of two scFv-CH3 chains are, with 80 kDa, about half the size of full antibodies. Furthermore, they lack a functional Fc-region, preventing extended circulation due to FcRn recycling. A recent study demonstrated the use of an anti-(human) PSCA minibody, called A11, conjugated with IRDye800CW in different mouse models. Subcutaneous, orthotopic and intramuscular implanted tumors and tumor positive lymph nodes could be clearly visualized, even in knock-in models expressing the human homolog of PSCA (Zhang et al., 2018). Furthermore, fluorescence-guided resection of intramuscular tumors, revealed superior overall survival compared to conventional white light surgery (Zhang et al., 2018).

However, the size of minibodies does still put them above the cut-off value for renal clearance. Compounds smaller than 60 kDa are generally cleared via the kidneys, resulting in faster blood clearance, and correspondingly, diminished non-specific uptake. High contrast should thus be attained at an earlier timepoint, though at the expense of total tumor uptake (total fluorescent signal) (Freise and Wu, 2015). It has furthermore been shown that an inverse relation exists between the compound's size and its rate of tissue penetration, meaning that smaller fragments will distribute more quickly and more deeply throughout tumor and healthy tissues (Li et al., 2016; Xenaki et al., 2017). Antibody fragment types that are under investigation for FIGS are scFv's (25 kDa), diabodies (scFv dimer, 50 kDa), and Fab fragments (single scFv-CH3 chain, 55 kDa). The PSCA-specific diabody A2cDb, site-specifically conjugated to Cy5 was evaluated in murine subcutaneous and intramuscular prostate cancer models and optimal imaging contrast was achieved within 6 h post-injection (Sonn et al., 2016). The same diabody, site-specifically conjugated to IRDye800CW and directly labeled with ^124^I, was recently used as bimodal tracer for the visualization of PSCA-positive pancreatic patient-derived orthotopic xenografts with PET and NIR fluorescence imaging. High-contrast imaging within 24 h or less was possible. It was furthermore demonstrated that the clearance pattern of the tracer was not significantly different from the iodinated-only form, indicating that the conjugation strategy used for IRDye800CW labeling did not impact the tracer's biodistribution or clearance (Zettlitz et al., 2018). This is in contrast to other studies with small antibody fragments that, as discussed above, often exhibit TBRs that are sufficiently high only between 24 and 72 h post-injection, as well as a significant amount of uptake in the liver. This has been found to be the case at least for an anti-HER3 diabody, anti-EpCAM-Fab fragment, and scFv's targeting CEA, EGFR and the tumor-associated glycoprotein 72 (TAG-72), all randomly labeled with IRDye800CW (Yang et al., 2014; Boonstra et al., 2015b; Alam et al., 2018; Gong et al., 2018; Boogerd et al., 2019).

In addition to the antibody fragments derived from conventional antibodies, the monomeric antigen-binding domains of heavy-chain only antibodies, so called single-domain antibodies or nanobodies, have been investigated for FIGS [recently reviewed in Debie et al. (2019)]. In preclinical studies, it has been shown that site-specifically IRDye800CW conjugated anti-HER2 and anti-CAIX nanobodies could achieve high contrast and specific imaging of tumor lesions within the first hours (1–4 h) after injection in murine breast and ovarian cancer models (Kijanka et al., 2013, 2016; Debie et al., 2017, 2018).

An advantage of antibodies and antibody-based tracers is that they can be generated in high through-put using established immunization strategies, and produced via fermentation in animal, yeast or bacterial cells (Ma and O'Kennedy, 2017). For tracer development, antibodies and antibody fragments represent a platform of compounds with a predominantly constant structure, except for variations in the antigen-binding loops prompting different specificities. This allows the definition of general properties and more versatile protocols for tracer preparation. Another type of platform technology are the non-antibody protein scaffolds that have a fixed backbone and are derived from different naturally occurring proteins. New antigen-specific protein scaffolds are selected from synthetic libraries and further engineered *in vitro*. As opposed to antibody-derived ligands, these compounds undergo no *in vivo* affinity maturation steps. Many different types of protein scaffolds have been described, but only affibodies, centyrins, and cystine knottins have been evaluated for FIGS so far.

**Figure 3:**
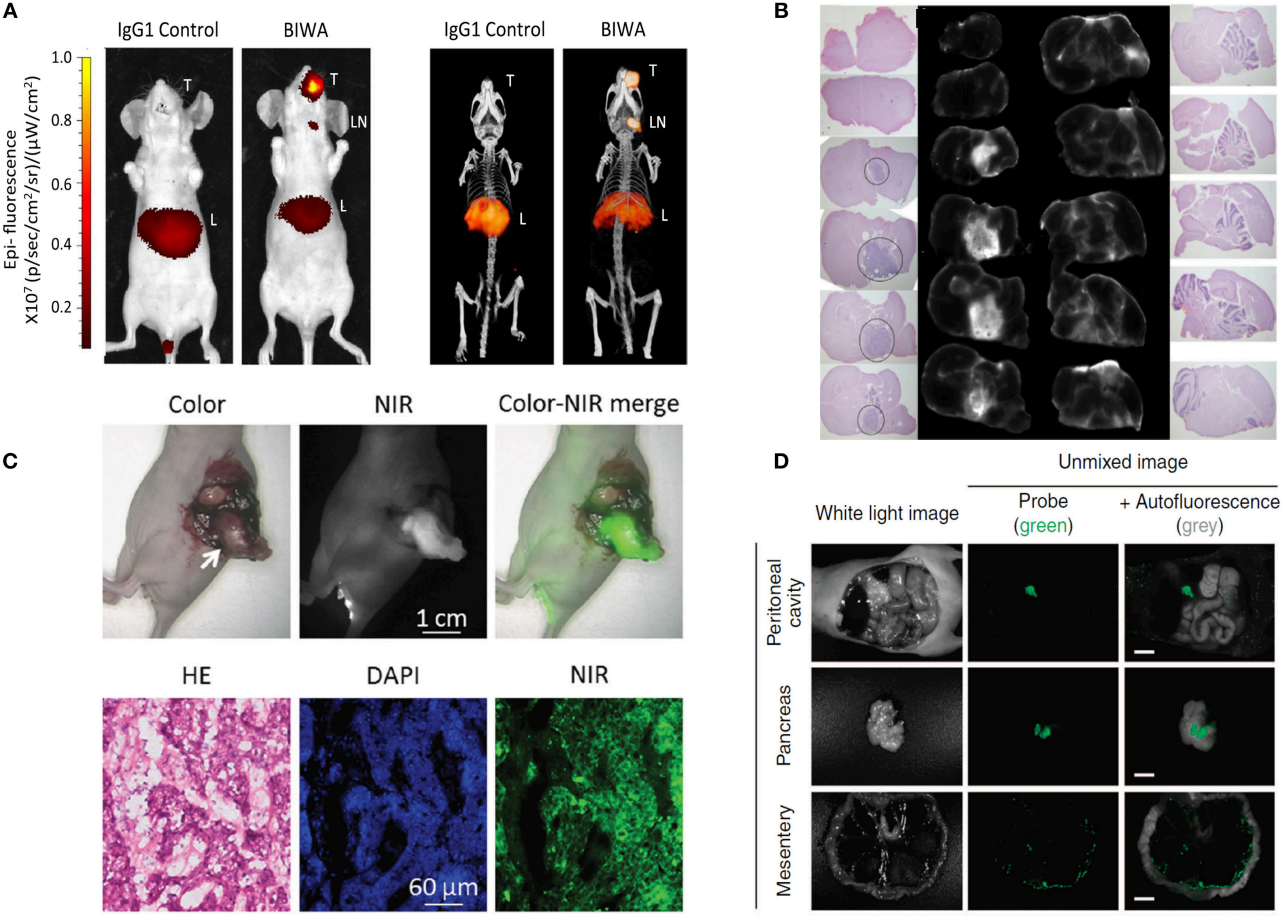
Examples of fluorescence imaging of tumor lesions with various preclinical FIGS tracers. **(A)** The anti-CD44v6 antibody BIWA co-conjugated with IRDye800CW and ^111^In 72 h after intravenous (iv) injection in an orthotopic CD44v6+ head and neck tumor model. Fluorescent signals (left) correspond well with results obtained via SPECT/CT imaging (right). Adapted from Odenthal et al. (2018) under the creative commons license. **(B)** Imaging of orthotopically implanted EGFR+ brain tumor slices after iv administration of an IRDye800CW conjugated anti-EGFR affibody. Tumor on hematoxylin and eosin stained slices co-localizes with high signal intensity on corresponding NIR images. Adapted from de Souza et al. (2017) under the creative commons license. **(C)** Imaging of an orthotopically implanted pancreatic cancer xenograft 4 h after iv injection of a ZW800-1 conjugated cRGD peptide. Specific tumor targeting by the tracer is confirmed by hematoxylin and eosin staining. Adapted from Handgraaf et al. (2017) under the creative commons license. **(D)** Imaging of abdominal ovarian cancer xenograft 5 min after intraperitoneal injection of a HMRef-βGal spirocyclic activatable tracer. Fluorescence becomes activated at the tumor site only. Adapted from Asanuma et al. (2015) under the creative commons license.

Affibodies are small proteins of about 7 kDa that have been derived from the B-domain of *Staphylococcus aureus* protein A. They consist of a three helical structure that can, by mutation of 13 amino acids in the first two helices, bind different molecular targets. The affibody tracer ABY-029, directed toward EGFR and conjugated site-specifically with IRDye800CW via a C-terminal cysteine, is currently undergoing clinical translation for FIGS in glioma, head and neck cancer and soft tissue sarcoma. This tracer was first evaluated in an orthotopic human EGFR-expressing glioma model in rats (Figure 3B). The tracer was found to preferentially accumulate in the tumor, and this from 1 h post-injection onwards (de Souza et al., 2017). Other studies using fluorescently-labeled affibodies targeting EGFR and HER2 show similar optimal imaging timepoints (Zielinski et al., 2012; Tummers et al., 2018a).

Centyrins are 10 kDa fibronectin type III domains, derived from human tenascin, made up of 7 antiparallel β-strands. Binding affinity against different targets is generated via mutations in the connecting loops. The human/mouse cross-reactive anti-EGFR centyrin 83v2 labeled with the fluorophore S0456, was preclinically tested in subcutaneous lung cancer xenografts. Imaging could be performed within the first hours after injection and peak fluorescence was reached between 6 and 12 h pi. (Mahalingam et al., 2017).

Cystine knot peptides (3–4 kDa) are built around a naturally occurring motif, where the peptide forms a type of knot stabilized by three cysteine-bridges around a core of antiparallel β-strands. Through mutations, the loops that connect the strands can confer specific binding capabilities to the knottin peptide. The knottin peptide Tozuleristide, which consists of the scorpion venom derived ligand chlorotoxin conjugated to ICG, is currently undergoing clinical translation. This probe was able to distinguish tumor from normal tissue in resected human breast cancer specimens between 1 and 26 h post-injection (Dintzis et al., 2018). Tozuleristide is a naturally occurring knottin, whose exact specificity is not fully known, though MMP-2 and AnnexinA2 have been observed as targets. Alternatively, the knottin peptide R01-MG, with engineered affinity for the human and mouse α_v_β_6_ integrin, has been tested pre-clinically. After conjugation with IRDye800CW, it was evaluated in subcutaneous, orthotopic, and spontaneous tumor models of pancreatic cancer. Fluorescent signal in a subcutaneous tumor became significantly different from a control knottin at 4 h post-injection, and maximal TBR was reached at 20 h post-injection. Cancer lesions, including small metastases could be clearly discerned from normal tissue in the orthotopic and spontaneous models (Tummers et al., 2018a).

Other than these scaffold proteins, non-scaffold peptides and small molecules can also be used as tracers in FIGS. The advantage of peptidic and small molecule-based tracers is that they can be produced at reduced cost compared to the other types of tracers. Target specific peptides and small molecules can be generated by rational design, or compounds with pre-existing affinities for the envisaged target, such as natural ligands, can be repurposed as fluorescent tracers. However, their structure and physicochemical properties vary greatly as they do not share a common backbone. General pharmacokinetic characteristics are hard to define, though peptide/small molecule-based tracers are expected to be small enough to attain at least partial renal clearance, depending on their hydrophobicity.

By far the most investigated peptide, is (cyclic) RGD, which targets a group of integrin receptors through a naturally occurring binding motif. cRGD peptides have been conjugated with a variety of different fluorophores, including Cy5.5, ICG, IRDye800CW and ZW800-1. After evaluation in subcutaneous and orthotopic tumor models of gastric, head and neck, pancreatic (Figure 3C) and colorectal cancer, tumor-to-background values were generally optimal between 4 and 24 h post-injection, depending on the fluorophore used (see above) (Choi et al., 2013; Cheng et al., 2016; Handgraaf et al., 2017). Other promising peptides for further development are tracers against for example PSMA (Wang et al., 2014; Bao et al., 2017; Baranski et al., 2017; Kularatne et al., 2018; Schottelius et al., 2018), the gastrin releasing peptide receptor (GRPR) (Cai et al., 2013; Suganya et al., 2016; Zhang et al., 2017a; Li et al., 2018; Xu et al., 2018) and uPAR (Yang et al., 2014; Christenen et al., 2017; Kurbegovic et al., 2018). Folate-receptor specific compounds were the first small molecules to be used in clinical trials for FIGS. The compound EC17 was constructed by conjugating folic acid with fluorescein isothiocyanate (FITC) (wavelength of maximal emission and excitation at 495/515 nm, outside the NIR region) (van Dam et al., 2011). EC17 was initially tested during debulking surgery in the case of late stage ovarian cancer, where FRα is widely expressed. To improve imaging contrast and detection of deeper located lesions, folic acid was labeled instead with the NIR dye S0456, to form OTL38. Additional clinical studies with both tracers have since been performed in lung, ovarian, and renal cancer. The surgical procedure is generally performed between 2 and 6 h post-injection of either tracer (Hoogstins et al., 2016; Keating et al., 2017; Mahalingam et al., 2018b; Predina et al., 2018).

## Activatable Tracers

An alternative to the conventional “always-on” targeted tracers, is the use of “activatable” or “smart” probes. The general idea behind this approach is that these are non-fluorescent until activation on their target site, thereby suppressing non-specific fluorescence signals and increasing tumor-to-background contrast (Lacivita et al., 2012). Two main types of activatable tracers can be distinguished, activatable targeted tracers and enzyme-activated probes (reviewed by Mochida et al., 2018).

Activatable targeted tracers are not dissimilar in structure to always-on tracers, meaning they also consist of a targeting moiety and conjugated fluorophore. The difference lies in the fact that as a result of the presence of a quencher or due to aggregation to the targeting moiety, fluorescence is quenched. Most often, the fluorescent groups only become activated after binding a molecular target on the cell surface, internalization into the targeted cell, and subsequent metabolization. The simplest construction of such a tracer is through the conjugation of ICG to a targeting moiety (Sano et al., 2013; Watanabe et al., 2014). ICG fluorescence has been found to be already significantly quenched at conjugation ratios of 1:1 with a monoclonal antibody. Upon cleavage in the lysosomes, ICG's full fluorescence activity is restored. It is however important to note that hepatic uptake, and consequent clearing of the antibody-ICG conjugate will cause significant background fluorescence (Ogawa et al., 2015). Alternatively, for an ATTO680-Trastuzumab conjugate, the use of a “molecular-switch” has been demonstrated (Kim et al., 2017). In this case, the fluorophore is initially quenched by interaction with tryptophan residues on an antibody's framework, and activated by conformational changes due to receptor binding. The dual labeling of an antibody with a fluorophore and a quencher is another possibility. This was demonstrated in a preclinical study, where the anti-EGFR antibody Cetuximab was co-conjugated with the fluorophore AF660 and quencher IRDye QC-1 at an average antibody:fluorophore:quencher ratio of 1:2:6. Similarly to ICG conjugated compounds, internalization into the target cells and subsequent catabolization will lift the quenching effect and reactivate fluorescence (Obaid et al., 2017).

Enzyme-activated tracers rely on the innate function of a target enzyme to activate the fluorescence of the probe. Proteases such as matrix metalloproteinases (MMPs), cathepsins, and β-galactosidases are often targeted by these tracers. Activation can occur in several manners. When probes are quenched because of the spatial proximity of the fluorophore to a quenching group (which can also be another fluorophore) cleavage of the tracer will separate the quenching group from the fluorophore and activate the fluorescence. Alternatively, the probe can form a spirocyclic structure caging the fluorophore, and only becoming active when it linearizes after enzymatic cleavage (Lacivita et al., 2012; Mochida et al., 2018). Two enzyme-activated tracers, called LUM015 and AVB-620 are currently being clinically tested. LUM015 is a probe consisting of a quencher, QSY21, linked to a Cy5 fluorophore using the peptide sequence “‘GGRK,” which is in turn also linked to a 20 kDa polyethylene glycol (PEG) on the Cy5 side. Upon cleavage by cathepsins, the Cy5 fluorophore regains its fluorescence. The PEG-chain is included into this structure to slow diffusion of the activated fluorophore and increase contrast. Indeed, if the probe is not retained, diffusion of signal from the cleavage site may negatively impact the potential for tumor delineation. Clinical studies in breast cancer and soft tissue sarcoma patients showed that tumor imaging could be performed between 2 and 30 h after intravenous injection of this tracer (Whitley et al., 2016; Smith et al., 2018). AVB-620 is an activatable cell-penetrating peptide. It consists of a Cy5 conjugated cell penetrating peptide, bound through a linker to a neutralizing, Cy7 conjugated, sequence. Due to the spatial proximity of both fluorophores, the Cy5 signal is quenched for the Cy7 fluorophore's emission. Upon cleavage by MMP-2 or MMP-9, both sequences are released, leading to cellular uptake of the Cy5 peptide (causing increased retention of fluorescence), increased Cy5 fluorescent signal and decreased Cy7 fluorescence. To differentiate tumor from normal, instead of the total fluorescent signal, the Cy5:Cy7 ratio is determined. In breast cancer patients, tumor delineation was generally possible between 2 and 20 h post-injection (Unkart et al., 2017). Other than these clinical studies, several other enzyme-activated tracers for FIGS are being preclinically developed (Table 2). Besides those that are similarly structured to the clinically investigated compounds (separate fluorophore and quenching group), several spirocyclic compounds may also be of interest for clinical translation. For example, a γ-Glutamyl-transpeptidase (GGT) activated probe called gGlu-HMRG was designed using the visible wavelength fluorophore hydroxymethyl rhodamine green (wavelength of maximal excitation/emission at 490/520 nm, outside the NIR region). Normally, this tracer is non-fluorescent due to quenching by its spirocyclic structure, but it becomes activated when the enzyme cleaves an internal γ-glutamyl bond and linearizes (Urano et al., 2011). This tracer, and similarly designed tracers have been tested on excised tumor tissue, and could differentiate tumor from normal after spraying on patient samples of colorectal, breast, oral, and ovarian cancer (Figure 3D) (Urano et al., 2011; Fujii et al., 2014; Asanuma et al., 2015; Sato et al., 2015; Ueo et al., 2015; Matsuzaki et al., 2016; Slooter et al., 2018).

**Table 2:** Comprehensive overview of potential activatable FIGS tracers.

**Tracer type**		**Compound name**	**Molecular target**	**Imaging target**	**Reporter fluorophore(s)**	**References**	**Remarks**
Activatables; Cell-binding based	Antibody	Cetuximab	EGFR	Pancreatic cancer^O^	AF660	(Obaid et al., 2017)	IRDye QC-1 quencher
		Trastuzumab	HER2	Lung cancer^S^	ATTO680	(Kim et al., 2017)	Molecular switch
		J591	PSMA	Prostate cancer^S^	ICG	(Nakajima et al., 2011)	
	Minibody	PSMA-Mb	PSMA	Prostate cancer^S^	ICG	(Watanabe et al., 2014)	
	Diabody	PSMA-Cys-Db	PSMA	Prostate cancer^S^	ICG	(Sano et al., 2013)	
	Peptide	EGF	EGFR	Breast cancer^S^, Skin cancer^S^	ATTO655, Cy5.5	(Ryu et al., 2013; Kim et al., 2019)	BHQ3 Quencher (Cy5.5)
Activatables; enzyme-activated		HMRef-bGal	β-Galactosidase	Ovarian cancer^Ip^	HMRef	(Asanuma et al., 2015)	Spirocyclic
		6QCNIR	Cathepsins	Breast cancer^O^, Lung cancer^Tg^	DyLight780-B1	(Ofori et al., 2015)	IRDye QC-1 quencher
		BMV109	Cathepsins	Colorectal cancer^Tg^	Cy5	(Segal et al., 2015)	QSY21 quencher
		GB119	Cathepsins	Glioma^O^, Skin cancer^Ev^	Cy5	(Cutter et al., 2012; Walker et al., 2017)	QSY21 quencher
		LUM015	Cathepsins	Breast cancer^C, O^, Soft tissue sarcoma^C, O^	Cy5	(Whitley et al., 2016; Smith et al., 2018)	Clinical trial, QSY21 quencher
		Z-Phe-Arg-HMRG	Cathepsins	Ovarian Cancer^Ip^	Hydroxymethyl rhodamine green	(Fujii et al., 2014)	Spirocyclic
		ANP_FAP_	FAP-α	Glioma^S^	Cy5.5	(Li et al., 2012)	QSY21 quencher
		gGlu-HMRG	GGT	Breast cancer^Ev^, Colorectal cancer^Ev^, Head and Neck cancer^O^	Hydroxymethyl rhodamine green	(Urano et al., 2011; Sato et al., 2015; Ueo et al., 2015; Slooter et al., 2018)	Spirocyclic
		HMRef-βGlcNAc	Hexosaminidase	Colorectal cancer^Ev, Ip^	HMRef	(Matsuzaki et al., 2016)	Spirocyclic
		Q_3_STCy	hNQO1	Ovarian cancer^Ev, Ip^	TCy	(Shen et al., 2017)	Q_3_ is target specific, and quencher
		AVB-620	MMP-1,9, …	Breast cancer^O^, Pancreatic cancer^O^	Cy5	(Metildi et al., 2015; Miampamba et al., 2017; Unkart et al., 2017)	Clinical trial, Cy7 quencher

^*^C, In clinical trial; Ev, Ex vivo human tumor samples; Ip, small animal intraperitoneally disseminated tumor model; O, small animal orthotopic tumor model; S, small animal subcutaneous tumor model; Tg, small animal transgenic spontaneous tumor model.

*EGFR, epidermal growth factor receptor; EpCAM, epithelial cell adhesion molecule; FAP-α, fibroblast activation protein alpha; GGT, gamma-glutamyltransferase; hNQO1, human NAD(P)H:quinone oxidoreductase type I; MMP-1/9, matrix metalloproteinase 1/9; PSMA, prostate specific membrane antigen*.

## Discussion on the Future Development of FIGS Tracers

Over the last decade, FIGS has been developing at a rising pace and the potential benefits of specific molecular tracers have become clear. Initial successes were reached with the clinical translation of the folate-based tracers EC17 and OTL38 as well as IRDye800CW-labeled antibodies. Meanwhile, additional tracers have entered the phase of clinical evaluation and many more are being tested at preclinical level; from classical antibody-based probes which are widely available and relatively easy to generate, to smaller tracers with higher *in vivo* specificity and faster kinetics allowing administration shortly before surgery. Novel designs are continuously emerging, and the importance of design-related aspects, such as the physicochemical properties of the fluorophore itself, the type of targeting ligand and the chosen target, have become apparent. Further research is needed to better understand how all these factors impact the *in vivo* pharmacokinetics and targeting of forthcoming FIGS tracers, to optimize their design and reduce their production cost.

Particular types of molecular tracers may furthermore be more suited for specific applications. For example, hybrid tracers are expected to offer superior sensitivity for the intra-operative detection of metastasized lymph nodes, while the ability to topically apply activatable tracers without the need for washing could be convenient to check the surgical wound for residual disease after resection or for the diagnosis of lesions during endoscopic examination.

Preclinical studies are valuable for initial assessment of the pharmacokinetics and possible application of FIGS tracers. It is, however, important to also critically appraise conclusions drawn from these studies, as there are limitations to the preclinical models used as indicators of possible future clinical success. Subcutaneous and orthotopic tumor models based on cell lines highly overexpressing the molecular target of interest are suitable for demonstrating specificity of the tracer, by comparing uptake with a non-targeting control compound or in receptor-negative cell line. However, although orthotopic models provide better insight on relevant tissue background signal, the homogenous manner of tumor development in these types of models does not adequately reflect the complexity and heterogeneity of a human tumor including its microenvironment. Furthermore, non-specific uptake of the tracer cannot be properly assessed unless it is reactive for the murine variant of the target as well, or alternatively, unless knock-in models of the relevant target are used. Although patient-derived xenografts can alleviate some of these issues, even using these advanced models, surgical efficiency remains difficult to assess in mice. Large animals may offer improvement as the more sizeable organs and inter-organ spaces of i.e., porcine and canine models make for a more realistic setting to assay FIGS contrast agents (Lwin et al., 2018a). Moreover, these can also be used to better estimate the required dose, monitor possible toxic effects, and even identify failure of the hypothesis before unnecessary large cost investments for cGMP drug development are made. In the end, however, the actual clinical value of new tracers can only be established in patients. This will in addition to the determination of merely objective endpoints such as safety, tumor negative margins, recurrence and survival, also enable the evaluation of more subjective outcomes such as quality of life.

## Author Contributions

PD and SH composed the manuscript together. Both authors critically revised and approved he manuscript for publication.

### Conflict of Interest Statement

SH holds a patent in camelid single-domain diagnostics and therapeutics. The remaining author declares that the research was conducted in the absence of any commercial or financial relationships that could be construed as a potential conflict of interest.
